# High-frequency radio wave electrocautery in modified Hotz operations for epiblepharon correction

**DOI:** 10.1186/s12886-021-02202-z

**Published:** 2021-12-10

**Authors:** Hyunkyu Lee, Jimin Youn, Sehyun Baek

**Affiliations:** 1 Department of Ophthalmology, Korea University College of Medicine, Ansan Hospital, Ansan, South Korea; 2 Department of Ophthalmology, Korea University College of Medicine, Guro Hospital, Seoul, South Korea

**Keywords:** Epiblepharon, Hotz operation, High-frequency radio wave electrocautery

## Abstract

To report the experience and advantageous effects of high-frequency radio wave electrocautery in modified Hotz operations for epiblepharon, We reviewed the records of all pediatric patients who underwent a modified Hotz operation with the use of high-frequency radio wave electrocautery (Ellman Surgitron Dual Frequency RF S5; Ellman International, Inc, Hewlett, NY) for epiblepharon between March 2016 and September 2019 at Korea University Guro Hospital. We evaluated the success rate, complications, recurrence rate and degree of satisfaction of our technique. Information from the medical records was collected, including demographics, ocular symptoms, severity of keratopathy, operation time, success/recurrence rate, and complications. 133 patients (98.52%) showed good correction of epiblepharon without complications or unpleasant cosmetic problems during 3 months of median follow-up period. Two patients (1.48%) showed recurrent corneociliary touch, but the degree was very mild and re-operation was not performed. One patient showed mild ectropion on his left lower eyelid, but the patient recovered well without operation. For complications, suture abscess and granulation were the most common, 3 cases in each, but all of those were temporary and resolved with conservative management. The approach with electrocautery for epiblepharon allows precise and fast incision of the lower eyelid, little bleeding, and minimal scarring. Surgical outcomes associated with the modified Hotz operation with electrocautery were consistent with previous studies.

## Background

Electrosurgical devices have developed since Dr. Albrecht Theodor Middeldorf first discussed and published application of electrical current in surgical interventions in 1854. Irving Ellman determined that a 4 MHz frequency electrocautery produced the smoothest cutting effects on tissue while limiting uncontrolled thermal damage [1]. Later, Stephen Bosniak introduced the radioelectrical device for his oculoplastic surgery approach in 1985. High-frequency radio wave electrocautery has since been used in various ophthalmology procedures, including punctoplasty, conjunctivochalasis correction, removal of lymphangiectasia or conjunctival cyst/chemosis, and eyelid surgery [2, 3]. A surgical approach with high-frequency radio wave electrocautery provides advantages, including effective hemostasis, shorter surgery time, and fast recovery [4].

Epiblepharon is defined as a horizontal fold of the skin and underlying orbicularis muscle that overrides the eyelid margin and pushes the lashes against the globe [5]. Corneociliary touch in epiblepharon causes keratopathy, and patients can experience tearing, photophobia, foreign body sensation, and visual disturbance [6]. Surgical treatment is pursued in severe cases with significant corneal injury. There are several surgical procedures including incisional and non-incisional techniques for treating epiblepharon [5, 7–10].

A modified Hotz operation is one of the most common surgery approaches for correcting epiblepharon. To date, a number of studies on modified Hotz operations has primarily focused on surgical outcomes such as success rate, recurrence, and complications [8, 11–13]. However, surgical instruments also can be a notable topic in modified Hotz operations. Appropriate surgical instruments allow surgeons to reduce efforts and to avoid unnecessary complications. Herein, we share the experience and advantageous effects of high-frequency radio wave electrocautery in modified Hotz operations for epiblepharon.

## Methods

**Fig. 1:**
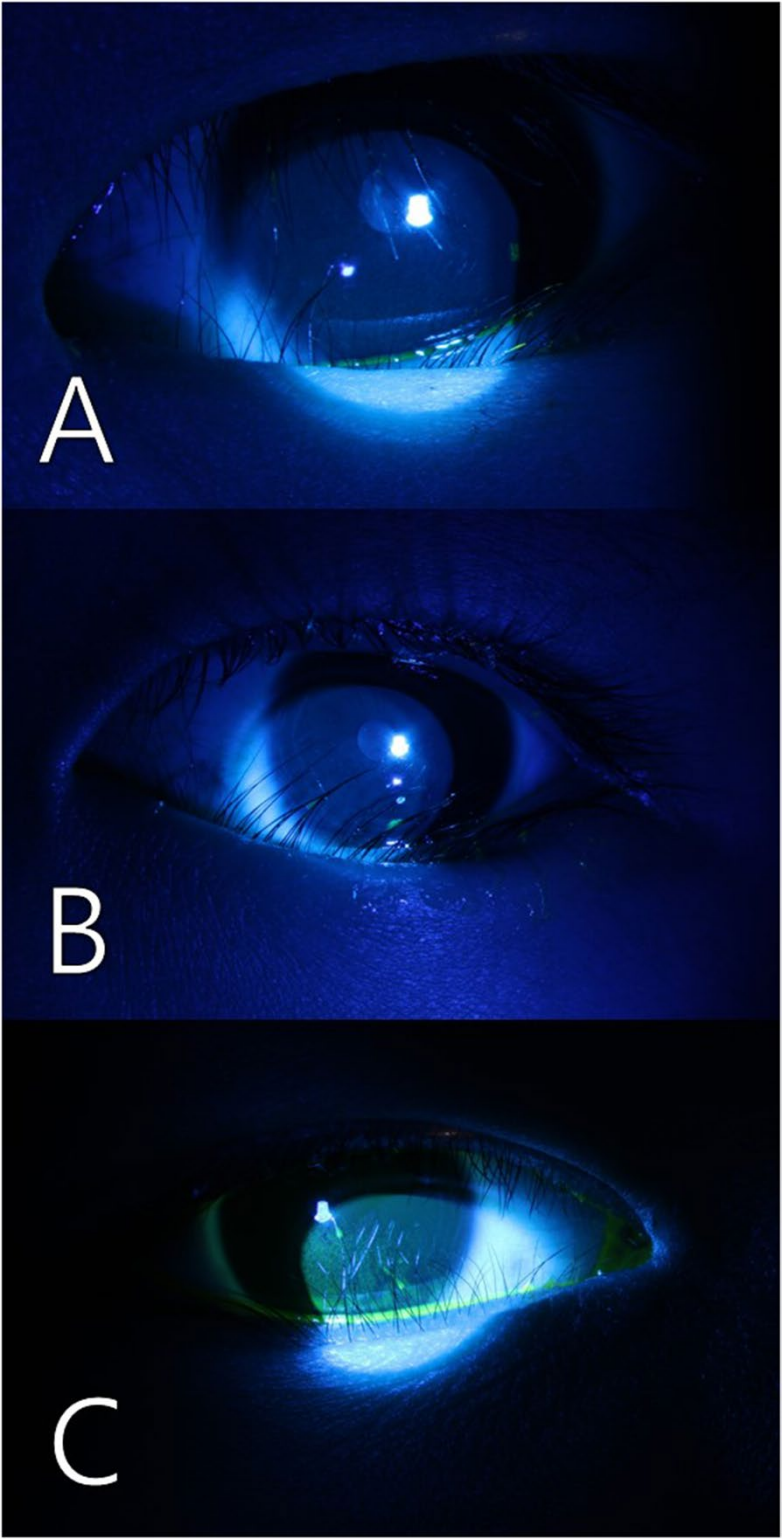
Keratopathy gradings caused by corneociliary touch in epiblepharon patients. (A) Mild, punctate, corneal erosions in an area that was less than the medial one-third of the cornea. (B) Moderate, punctate, corneal erosions in an area greater than the medial one-third of the cornea. (C) Severe, punctate, corneal erosions in an area greater than the medial one-third of the cornea with patches of confluent corneal staining.

This was a retrospective chart review of all pediatric patients who underwent a modified Hotz operation with the use of high-frequency radio wave electrocautery (Ellman Surgitron Dual Frequency RF S5; Ellman International, Inc, Hewlett, NY) for epiblepharon between March 2016 and September 2019 at Korea University Guro Hospital. Information from the medical records was collected, including demographics, ocular symptoms, severity of keratopathy, operation time, success/recurrence rate, and complications. Keratopathies were classified as mild, moderate, and severe: mild for punctate corneal erosions in an area less than the medial one-third of the cornea, moderate for punctate corneal erosions in an area greater than the medial one-third of the cornea, and severe for punctate corneal erosions in an area greater than the medial one-third of the cornea with patches of confluent corneal staining (Fig. 1). Moderate and severe keratopathy were considered significant findings in terms of treatment.

A total of 135 pediatric patients under 15 years of age was included in this study. The surgery was indicated when patients showed significant keratopathy and/or irritation symptoms, and the procedure was performed by a single surgeon (S.B.). All subjects were followed for more than one month. The protocol for this study was approved by the Institutional Review Board of Korea University Guro Hospital. Consent was obtained from each patient for use of identifying photographs.

**Fig. 2:**
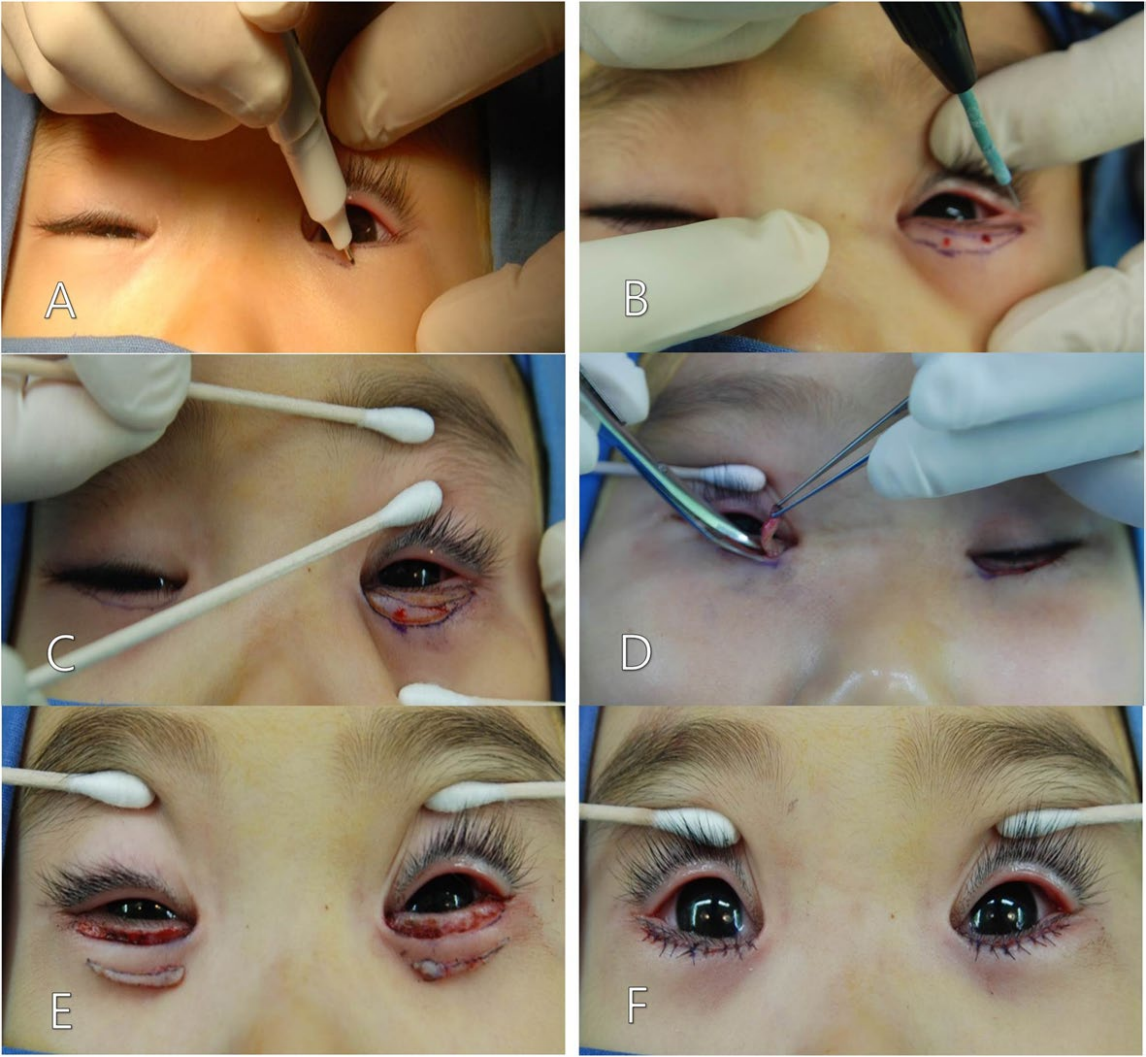
Surgical procedures for the modified Hotz operation. (A) A subciliary incision line on the lower eyelid was marked with pen. (B, C) High-frequency radio wave electrocautery was used to incise the skin of the lower eyelid along the elliptical design. (D, E) Redundant skin and muscle were excised with Westcott scissors, and a strip of pretarsal orbicularis oculi muscle was removed with a hand cautery. (F) Skin was closed with a 6-0 vicryl interrupted suture.

The procedures were performed under general anesthesia. After the surgeon marked the subciliary incision line on the lower eyelid, subcutaneous infiltration of the lower eyelid with half-and-half by volume of lidocaine 2% with 1:200,000 epinephrine and bupivacaine 0.5% was performed. High-frequency radio wave electrocautery was applied (cutting mode, setting power: 13, fine needle tip), and the skin of the lower eyelid was incised along the design with quick fluid motions to minimize thermal tissue damage. Redundant skin and muscle were excised with Westcott scissors, and a strip of pretarsal orbicularis oculi muscle was removed with a hand cautery (Accu-Temp High Temperature Cautery, Medtronic Inc., Minneapolis, MN). Then, skin was closed with a 6-0 vicryl interrupted suture (Fig. 2).

## Results

The demographic characteristics of the 135 subjects (59 male, 76 female) are shown in Table 1. The average age at time of surgery was 6.3 years. None of the patients had undergone previous surgery on their lower eyelids. Four patients who underwent a combined operation, 1 for a double fold and 3 for Monoka tube intubation, were also included. All patients showed more than moderate to severe keratopathy in at least one eye. For all patients, a modified Hotz operation was performed on both eyelids to accomplish satisfactory results in terms of symmetry.

**Table 1:** Surgical outcomes of modified Hotz operation with high frequency radio-wave electrocautery

Outcomes	No. of cases
Success rate (%)	133 (98.5)
Recurrence rate (%)	2 (1.5)
**Complications (%)**	
Suture abscess	3 (2.2)
Granulation	3 (2.2)
Overcorrection	1 (0.7)
Undercorrection	None
Follow-up (months, range)	3 (1-44)
Surgery time (minutes, range)	20 (18-25)

Of the total 135 patients, 133 (98.52%) showed good correction of corneo-ciliary touch. The recurrence rate was 1.48% (2/135) during the 3-month median follow-up period. The average operation time was 20 minutes. Cases of undercorrection were not noted, but there was one case of overcorrection. Ectropion was mild, and the eyelid was returned to its normal position with conservative massage. There were three cases of suture abscess and three cases of granulation. All complications were temporary and resolved with conservative treatment (Table 1).

## Discussion

Epiblepharon is defined as a horizontal fold of skin and underlying orbicularis muscle that override the eyelid margin and push the lashes against the globe [5]. Noda et al. reported that mild epiblepharon can be treated with a conservative approach, after which the condition can spontaneously improve over time [14]. However, severe corneo-ciliary touch can cause prompt corneal injury and can later result in astigmatism and even higher-order aberrations [15, 16]. In this context, Tan P indicated that establishing a common objective system for grading epiblepharon is advisable to help determine the appropriate indications for surgery and has introduced studies related to epiblepharon grading [17–19]. In our study, surgery was indicated when the condition caused significant keratopathy and/or persistent irritative symptoms.

Several approaches can be considered when surgical treatment is indicated, including everting sutures, simple lid bracing sutures, cilia rotation sutures, and modified Hotz procedures [5, 7–10]. Hotz introduced a new technique for entropion and trichiasis for the upper eyelid in 1879. Since this introduction, a modified Hotz operation for the lower eyelid has been one of the most common surgeries for correcting epiblepharon. Sundar et al [11] reported an 83% success rate associated with the modified Hotz operation and noted better results for recurrence compared with lid-everting sutures. Woo and Kim [10] discussed the risk of ectropion or eyelid retraction from excessive skin excision with the modified Hotz operation in epiblepharon repair. We recognized the risk of overcorrection in the modified Hotz operation and have been careful with our approach to not remove excess skin. In our study, there was only one case of mild ectropion (0.7%), and the patient recovered at 1 month after conservative treatment.

High-frequency radio wave electrocautery can be used in various ophthalmology procedures including punctoplasty, conjunctivochalasis correction, removal of lymphangiectasia or conjunctival cyst/chemosis, and eyelid surgery [2, 3]. This electrocautery can be utilized for cutting, hemostasis, dissection, and dehydration. The electrocautery cuts the tissue with simultaneous hemostasis, which is very useful in the modified Hotz operation because the eyelid has a rich vascular supply. Reduced bleeding maintains a clear surgical field and allows the surgeon to perform quick and precise incisions along the marking line. In our study, the average operation time was 20 minutes for 131 patients, with the exception of 4 patients who underwent a combined operation. This may not be the case for all experienced surgeons, but electrocautery can be beneficial particularly for novice surgeons that may want to take more time and may be cautious with their approach. Moreover, high-frequency radio wave electrocautery can provide favorable results especially in regard to scarring after surgery. Bridenstine [3] shared his experience with electrocautery and indicated that patients reported rapid healing and aesthetically pleasing scars in various surgeries, including eyelid surgery. Though the surgery for epiblepharon is performed to correct a pathologic condition, the parents of pediatric patients often are concerned about aesthetic results. In our surgical approach with the modified Hotz operation using high-frequency radio wave electrocautery, postoperative bleeding or bruising events were not noted, and associated scarring complications were not reported.

In conclusion, there are many advantages to using high-frequency radio wave electrocautery in a modified Hotz operation for epiblepharon. The approach with electrocautery for epiblepharon allows precise and fast incision of the lower eyelid, little bleeding, and minimal scarring. Surgical outcomes associated with the modified Hotz operation with electrocautery were consistent with previous studies.

## Data Availability

The datasets used and/or analysed during the current study are available from the corresponding author on reasonable request.
